# Management of Tuberculosis: Are the Practices Homogeneous in High-Income Countries?

**DOI:** 10.3389/fpubh.2020.00443

**Published:** 2020-09-04

**Authors:** Frédéric Méchaï, Hugues Cordel, Lorenzo Guglielmetti, Alexandra Aubry, Mateja Jankovic, Miguel Viveiros, Miguel Santin, Delia Goletti, Emmanuelle Cambau

**Affiliations:** ^1^APHP, Infectious Disease Unit, Avicenne Hospital, Université Paris 13, IAME, INSERM, Bobigny, France; ^2^APHP, Groupe Hospitalier Universitaire Sorbonne Université, Hôpital Pitié-Salpêtrière, Centre National de Référence des Mycobactéries et de la Résistance des Mycobactéries aux Antituberculeux, Paris, France; ^3^Sorbonne Université, INSERM, U1135, Centre d'Immunologie et des Maladies Infectieuses, Cimi-Paris, Paris, France; ^4^Clinic for Lung Diseases, University of Zagreb School of Medicine and University Hospital Center Zagreb, Zagreb, Croatia; ^5^Global Health and Tropical Medicine, GHTM, Instituto de Higiene e Medicina Tropical, IHMT, Universidade Nova de Lisboa, UNL, Lisboa, Portugal; ^6^Service of Infectious Diseases, Tuberculosis Unit, Bellvitge University Hospital-IDIBELL, University of Barcelona, L'Hospitalet de Llobregat, Barcelona, Spain; ^7^Translational Research Unit, Department of Epidemiology and Preclinical Research, “L. Spallanzani” National Institute for Infectious Diseases (INMI), IRCCS, Rome, Italy; ^8^AP-HP, Hôpital Lariboisière, Service de Bactériologie, Paris, France

**Keywords:** tuberculosis, survey, Europe, guidelines, harmonization, diagnosis, treatment

## Abstract

**Objectives:** To evaluate and compare practices regarding the diagnosis, isolation measures, and treatment of tuberculosis (TB) in high-income countries and mainly in Europe.

**Materials and Methods:** A survey was conducted from November 2018 to April 2019 within the European Society of Clinical Microbiology and Infectious Diseases Study Group for Mycobacterial Infections (ESGMYC). The practices observed were compared to the main international guidelines.

**Results:** Among 136 ESGMYC members, 64 (17 countries) responded to the questionnaire. In their practice, two (20.7%) or three sputum samples (79.3%) were collected for the diagnosis of pulmonary TB, alternatively induced sputum (*n* = 37, 67.2%), bronchoscopy (34, 58.6%), and gastric aspirates (15, 25.9%). Nucleic acid amplification tests (NAATs) were performed by 41 (64%) respondents whatever the smear result and by 47 (73%) in case of smear-positive specimens. NAAT and adenosine deaminase measurement were used for extrapulmonary TB diagnosis in 83.6 and 40.4% of cases, respectively. For isolation duration, 21 respondents (42.9%) were keeping isolation until smear negativity. An initial treatment without ethambutol was offered by 14% (*n* = 9) of respondents. Corticosteroid therapy, cerebrospinal fluid opening pressure testing, and repeated lumbar puncture were carried out for central nervous system TB by 79.6, 51.9, and 46.3% of the respondents, respectively. For patients with human immunodeficiency virus–TB coinfection, the preferred antiretroviral therapy included dolutegravir 50 mg twice a day (56.8%). Comparing with the recommendations of the main guidelines, the practices are not totally consistent.

**Conclusion:** This study shows heterogeneous practices, particularly for diagnosis, and isolation, although rapid molecular testing is implemented in most centers. More standardization might be needed.

## Introduction

In 2017, 10 million people were diagnosed with tuberculosis (TB) in the world (1, 2). Although the European region accounted for only 3% of all cases (3), TB remains a common infection. Despite a rate of latent TB estimated at 23% of the world population, there are very large disparities in incidence between continents and countries with an incidence of fewer than 10 per 10,000 inhabitants in Western Europe to more than 500 per 100,000 for countries such as South Africa, the Philippines, and Mozambique (2). The overall objective of the World Health Organization (WHO) by 2035 (The End TB strategy) is to reduce the number of deaths from TB by 95% (compared to 2015) and to reduce the incidence rate of TB by 90% to fewer than 10 per 100,000 people. In 2016, 58,994 cases of TB were reported in 30 European Union/European Economic Area (EU/EEA) countries (2). The decreasing notification rates observed in most countries are reassuring, but annual rates of decline are still insufficient to achieve the WHO target of TB elimination by 2050 in European low-incidence countries. Multidrug-resistant (MDR) TB was reported for 3.7% of 36,071 cases with drug susceptibility testing results and continues to be the highest (more than 10%) in the three Baltic countries. Extensively drug-resistant (XDR) TB was reported for 20.1% of 984 MDR TB cases tested for second-line drug susceptibility (4). Data on human immunodeficiency virus (HIV) coinfection remained widely incomplete in Europe. Of all TB cases with known HIV status, 4.5% were coinfected with the virus (5).

An evaluation of TB case management in the EU/EEA countries, with special focus on MDR and XDR-TB, was conducted in 2010, using a standardized survey tool in five European centers. Deviations from international standards of TB care were observed in the following areas: surveillance (no information available on patient outcomes); infection control (lack of respiratory isolation rooms/procedures and negative-pressure ventilation rooms); clinical management of TB, MDR-TB, and HIV coinfection (inadequate bacteriological diagnosis, regimen selection, and treatment duration); laboratory support; and diagnostic/treatment algorithms (6).

A response to this need of harmonization has already been initiated through the development of European Union Standards for TB Care (ESTC) in 2012 with an update in 2017. They identified key standards for the diagnosis, management, prevention, and control of TB, MDR-TB, and XDR-TB. These standards aim to support health care workers in optimizing TB case management and thus at contributing to improved TB control in the EU/EEA (7–11). In fact, after the implementation of these standards, the knowledge of appropriate TB case management in high-income countries and in particular the different European countries is scarce. The main aim of this study was therefore to verify the current good homogeneity of practices of the different actors involved in the fight against TB and to compare them with the main national or international recommendations.

## Materials and Methods

A survey was conducted from November 2018 to April 2019 among members of the European Society of Clinical Microbiology and Infectious Diseases (ESCMID) Study Group for Mycobacterial Infections (ESGMYC), an offshoot of the ESCMID. The questionnaire was initially sent out on November 8, 2018, with subsequent reminders, the last being sent on March 15, 2019. The online questions comprised 63 items and focused on three topics: (1) diagnosis of TB, (2) isolation and prevention of TB transmission, and (3) TB treatment (summarized in Table 1). Descriptive statistics were used to analyze the results of the survey. The data were analyzed using STATA 10.0 (StataCorp LP, College Station, TX, USA). Paris-Seine-Saint-Denis University Hospitals Committee review waived the requirement for ethical approval for this study because of absence of interventional research on the human person, in accordance with the national legislation and the institutional requirements.

**Table 1:** Summary of the questionnaire.

**Respondents**	Name, country, institution, age, profession
	Medical information used for the practice
**Microbiological TB diagnosis**	Investigations for a suspicion of active pulmonary tuberculosis
	Management of NAAT for pulmonary TB
	Microbiological monitoring
	Management of NAAT for non-pulmonary samples
	Criteria for using ADA
	TB meningitis: diagnosis and management of corticosteroids and rifampicin
	Criteria for using IGRA and TST
**Isolation practices**	Kind of isolation room
	Duration of isolation and circumstances to stop isolation
	Isolation criteria of smear negative patients
	Hospital discharge criteria
**TB treatment**	Standard treatment of tuberculosis
	Fluoroquinolones treatment criteria
	Ethambutol if no isoniazid resistance mutation
	B6 vitamin treatment criteria
	Ophtalmology exam and ethambutol
	Drug blood levels following TB treatment
	HIV treatment in case of TB coinfection
	Management of latent TB in HIV patients
	Management of TB compliance

*NAAT, Nucleic acid amplification tests*.

In a second step, in order to understand the differences in the management of TB in high-income countries, we compared the participants' responses to the main principles of TB management according to the following international recommendations: Infectious Disease Society of America/Centers for Disease Control/American Thoracic Society (IDSA/CDC/ATS) guidelines (12, 13), National Institute for Health and Care Excellence (NICE) guidelines (14), ERS/ECDC (European Respiratory Society/European Centers for Disease Control) guidelines (11), WHO (15), and the recommendations of the French Hospital Hygiene Society (16). These are summarized in Tables 2–4 for comparing the proposals of each academic society.

**Table 2:** Microbiological TB diagnosis: synthesis of the international recommendations and main results from the ESGMYC survey.

**Diagnostic tools**	**IDSA/ATS/CDC 2017 (12)**	**NICE 2016 (14)**	**WHO Europe 2017 (17)**	**ERS/ECDC 2017 (11)**	**ESGMYC survey (% of respondents)**
**Pulmonary TB**	• Three sputum specimens • Sputum volume of at least 3 ml (optimal 5–10 ml) • Both liquid and solid culture (at least one liquid culture on all specimens)	• Three sputum specimens • Preferably one early morning sample	• Three sputum specimens • Both liquid and solid culture	• At least two sputum specimens (at least one early morning) for microscopic examination • Samples can be collected on the same day • Good quality sputum • At least liquid culture • Phenotypic drug susceptibility testing (DST)	• Sputum collection in the early morning (97%) • Three samples (81%) • 3 consecutive days (58%)
**Other procedures (patient unable to expectorate)**	• Sputum induction rather than flexible bronchoscopy sampling • Collect post-bronchoscopy sputum specimens	• Three sputum inductions or Three gastric lavages • Induction of sputum or bronchoscopy and lavage in adults	NA	• Sputum induction • Bronchoscopy/bronchoalveolar lavage • Gastric lavage in children	• Induced sputum (66%) • Bronchoscopy (59%) • Gastric aspiration (27%)
**Molecular tests (pulmonary TB)**	• NAAT on the initial specimen • A rapid molecular drug susceptibility testing for rifampicin with or without isoniazid • Genotyping in regional laboratory for each mycobacterial culture-positive	• NAAT on primary specimens if clinical suspicion of TB disease and/or • HIV-positive patient, or • Rapid information about mycobacterial species would alter the person's care, or • Need for a large contact tracing initiative is being explored.	• NAAT on at least one specimen	• One sputum tested for NAAT • WHO-recommended rapid molecular assays (sputum sample) • For DST, if use of WGS, results must be confirmed by phenotypic testing.	• Systematically done (64%) • Detection of resistance mutations to rifampicin (84%), isoniazid (29%), and others drugs (7%) • Mutations sought for INH resistance were *katg* gene and *InhA* promoter (64%), or only *katG* gene (15%) • Systematically whole genome sequencing (19.4%)
**Extrapulmonary TB**	• Cell counts and biochemistry on fluid specimens • ADA on fluid from pleural TB, meningitis, peritoneal, or pericardial TB • Free IFN-γ on fluid from suspected pleural or peritoneal TB • Smear microscopy for suspected extrapulmonary TB • NAAT from sites of extrapulmonary TB • Histological examination	• Addition of a spontaneously produced respiratory sample • Pleural TB: ADA, sputum samples and pleural biopsy • Meningeal TB: CSF fluid analysis, ADA, and NAAT • Lymph node: biopsy (+ histology), aspirate (+ cytology), NAAT • Pericardial TB: biopsy of pericardium (+histology), pericardial fluid (+ cytology), NAAT, ADA • Disseminated TB: biopsy (lung, liver, and bone marrow), bone marrow aspirate, bronchial lavage, appropriate CSF tests, and blood culture	NA	• Tests recommended for all cases: microscopy, NAAT, and culture, histopathology	• NAAT (85%) • ADA (40%): on pleural fluid (96%), CSF (40%), and peritoneal fluid (24%)

*NAAT, Nucleic acid amplification tests; WGS, whole genome sequencing; ADA, adenosine deaminase; TB, tuberculosis; IFN-γ, interferon-gamma; CSF, cerebrospinal fluid; DST, drug susceptibility testing*.

**Table 3:** Isolation measures recommended for pulmonary tuberculosis patients in healthcare settings: synthesis of international recommendations and ESGMYC survey main results.

**Isolation measures**	SF2H^*^ 2013 (16)	**ATS/CDC/IDSA 2005 (18)**	**NICE 2016 (14)**	**ERS/ECDC 2017 (11)**	**ESGMYC Survey (% of respondents)**
Smear-positive pulmonary TB cases	Up to 2 weeks if: • No suspicion of resistance • Decrease of cough intensity • Low initial smear grade • No immunocompromised patients in the same ward	Isolation until: • Patient under standard multi drug anti TB therapy, • Clinical improvement and • Three consecutive AFB-negative smear results of sputum specimens	Up to 2 weeks if: • (Complete) adherence to treatment • Resolution of cough • Improvement on treatment • Low initial smear grade (2 or less) • No cavitation • No laryngeal TB	Until: Bacteriological conversion (negative sputum smear)	Duration: • Standardized duration of 2–3 weeks (36%) • Until bacteriological conversion (36%)
Smear negative tuberculosis cases, suspicion of pulmonary TB	Isolation until culture results are available	NA	NA	NA	No isolation (28%)
Type of room for hospitalization	NA	NA	Single room/negative pressure room for patients at high risk of MDR-TB	Negative pressure ventilation room	Negative pressure room (77%)
Type of ward	NA	NA	No admission in wards with immunocompromised patients	NA	NA
Conditions of discharge from hospital	NA	Non infectiousness if: • Negligible likelihood of MDR TB • Anti TB therapy for 2–3 weeks • Complete adherence to treatment • Clinical improvement (reduction of cough, or of the grade of the sputum AFB smear result • Close contacts identified	• No continuing clinical or public health need for admission • unlikely to be rifampicin resistant • To avoid congregate settlings in the first 2 weeks of treatment	NA	To leave the hospital in case of positive smear sputum (75%) pending of conditions • Mask (65%) • No other people at home (81%) • Absence of children at home (60%) or • No immunocompromised people (75%) at home

MDR-TB, multidrug-resistant tuberculosis.

* *French Society of Hospital Hygiene*.

**Table 4:** Drug-susceptible tuberculosis treatment: synthesis of international recommendations and ESGMYC survey main results.

**Treatment scheme**	**IDSA/ATS/CDC 2017 (13)**	**NICE 2016 (14)**	**WHO 2010/2017 (15)**	**ERS/ECDC 2017 (11)**	**ESGMYC survey (% of respondents)**
Intensive phase	• INH/RIF/PZA/EMB • Stop EMB if susceptibility to RIF and INH	INH/RIF/PZA/EMB for 2 months	INH/RIF/PZA/EMB for 2 months	INH/RIF/PZA/EMB for 2 months	• INH/RIF/PZA/EMB (81%) • Tritherapy without ethambutol (14%)
Continuation phase	INH/RIF	INH/RIF for 4 months	INH/RIF for 4 months	INH/RIF for 4 months	NA
Doses	• Intensive phase: 7 days/week for 56 doses or 5 days/week for 40 doses^*^ • Continuation phase: 7 days/week for 126 doses or 5 days/week for 90 doses^**^	• Fixed-dose combination tablet • Dosing regimens of fewer than three times per week are not recommended • Daily dosing schedule is recommended	• Fixed-dose combination tablet • Daily dosing schedule is recommended	• Daily dosing schedule is recommended	NA
Adjunctive pyridoxine	To all persons at risk of neuropathy^***^	During all the treatment	NA	NA	Systematically for 89% of respondents
HIV co-infection	• 6 months treatment duration • 9 months if no antiretroviral therapy • Caution with drug–drug interactions	• 6 months duration • Caution with drug–drug interactions	• 6 months duration • Start of antiretroviral treatment within 8 weeks (within the first 2 weeks if CD4 counts <50 cells/mm^3^)	Delay between the initiation of TB therapy and the start of antiretroviral treatment of at least 14 days	• Associated antiretroviral therapy: dolutegravir 50 mg bid (60%), efavirenz 600 mg daily (35%), a protease inhibitor with rifabutin (17%), raltegravir 800 mg bid (15%), raltegravir 400 mg bid (13%), and efavirenz 800 mg daily (6%). • Preventive treatment for all HIV-positive patients with latent TB infection (100%)
Adjunctive corticosteroids	• TB pericarditis: not to be used routinely (if large effusions, high levels of inflammatory cells or markers, early signs of constriction) • Central nervous system TB: dexamethasone or prednisolone for 6–8 weeks	• Central nervous system TB and active pericardial TB: dexamethasone (CNS) or prednisolone (CNS, pericardial)	• TB meningitis: dexamethasone or prednisone first 6–8 weeks • TB pericarditis: initial adjuvant corticosteroid therapy may be used (conditional recommendation)	• TB meningitis: dexamethasone or prednisone during the first 6–8 weeks of treatment • TB pericarditis • Renal TB: to prevent ureteric stenosis • Spinal TB: if spinal cord compression	• Use of corticosteroids for neuro-meningeal TB (80%) • Duration of treatment with corticosteroids from 2 (10%) to 12 weeks (5%)
Adjunctive surgery	NA	• Central nervous system TB with raised intracranial pressure • Spinal TB with spinal instability or spinal cord compression	NA	NA	NA
Tuberculosis of the central nervous system	• INH/RIF/PZA/EMB for 2 months then INH/RIF 7–10 months • Repeated lumbar punctures	INH/RIF/PZA/EMB for 2 months (with pyridoxine) then INH/RIF 10 months (with pyridoxine)	NA	NA	• Measurement of CSF pressure (51%) • Repeated lumbar punctures (46%)
Culture negative tuberculosis	• Bronchoscopy with bronchoalveolar lavage and biopsy • Start treatment •Duration 4 months if clinical or radiographic response after 2 months of intensive phase therapy	Start treatment without waiting for culture results if life-threatening disease	NA	NA	NA
Response to therapy	NA	NA	Follow up smear microscopy at time of completion of the intensive phase and 5–6 months if initial smear positive	Follow up smear microscopy and culture at least at time of completion of the intensive phase	Sputum smear repeated after: • 2 months (83%) • 6 months (58%) of treatment
Optic neuritis	Visual acuity and color discrimination tests monthly during EMB use	NA	NA	NA	Ophthalmology assessment during the first 2 months of ethambutol treatment (50%)
Therapeutic Drug Monitoring	• Suspicion of drug malabsorption, drug underdosing, and clinically important drug-drug interactions • Delayed sputum conversion or treatment failure • Reduced renal function • Treatment for drug-resistant tuberculosis	NA	NA	Therapeutic drug monitoring if poor response to treatment (underdosing or malabsorption)	Therapeutic drug monitoring for rifampicin and isoniazid: • Never (50%) • Routinely (17%) • Only in specific cases (23%)
Management of IRIS	• Mild IRIS: TB and HIV treatment continued, and anti-inflammatory drugs • Severe IRIS: drainage for pleural effusions or abscesses, corticosteroids (2–4 weeks of treatment with subsequent tapering over a period of 6–12 weeks or longer)	NA	NA	NA	NA
Recurrence	• Retreatment using the standard intensive phase regimen • Rapid molecular tests to detect resistance	NA	• Drug-susceptibility testing before the start of treatment • Rapid molecular tests if possible	NA	NA
Adherence	Suggest using DOT	NA	• Health education and counseling (adherence interventions) • DOT (or VOT: Video observed Treatment)	NA	Interpreter use (56%) Nurse specialized in therapeutic education (48%) Systematic DOT (47%) Hospitalization for the full duration of treatment (28%) DOT at home with a nurse (61%)

INH, isoniazid; RIF, rifampicin; PZA, pyrazinamide; EMB, ethambutol; TB, tuberculosis; CNS, central nervous system; IRIS, immune reconstitution inflammatory syndrome; DOT, directly-observed therapy.

* Preferred regimen.

** Extend the continuation phase for patients with cavitation on the initial chest radiography and patients who have culture positive after completion of 2 months of therapy.

*** *Pregnant women; breastfeeding infants; persons infected with human immunodeficiency virus [HIV]; patients with diabetes, alcoholism, malnutrition, or chronic renal failure; or those who are of advanced age*.

## Results

### Respondents

**Figure 1:**
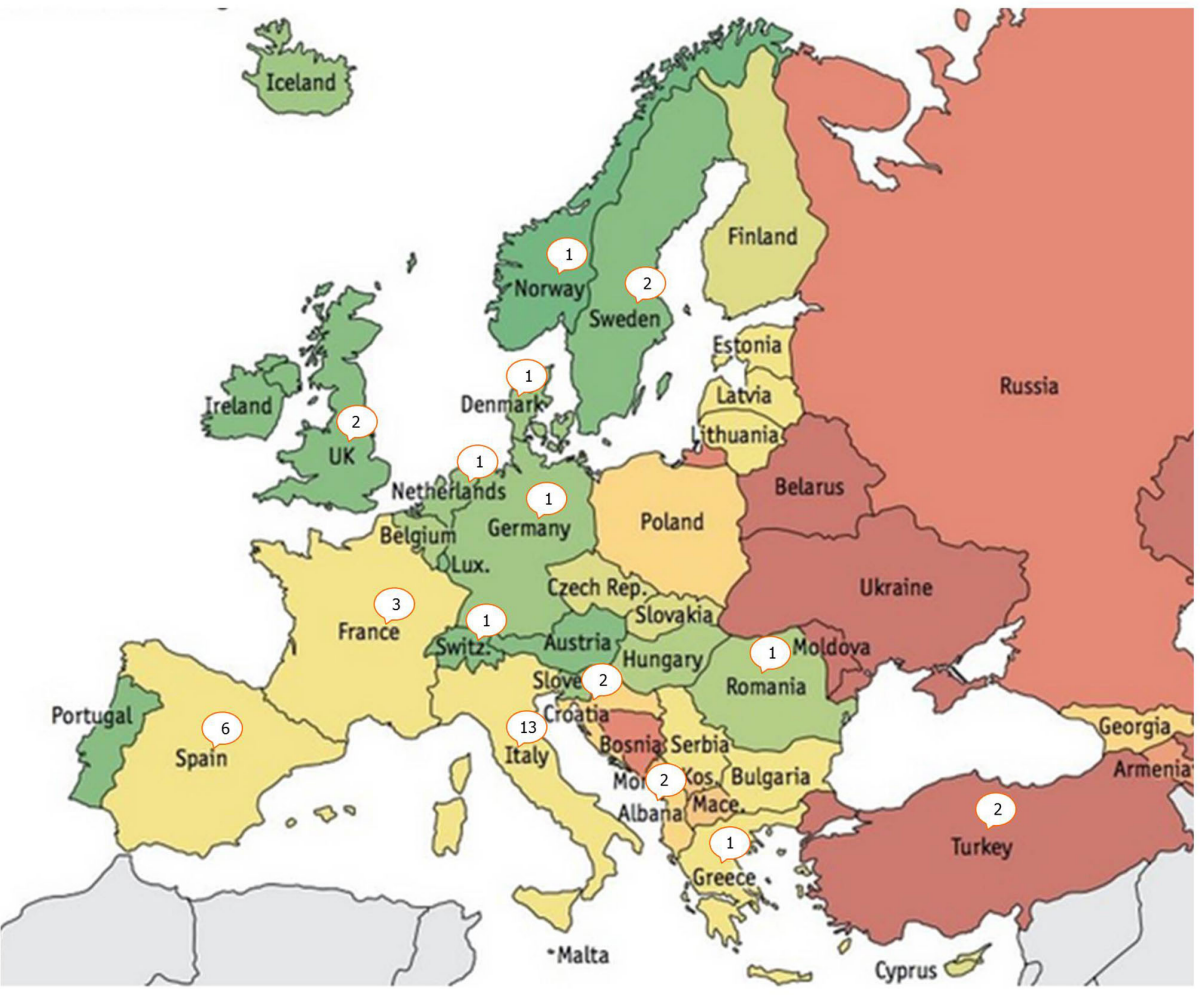
Europe map presenting the number of respondents per country.

Among 136 ESGMYC members, 64 (47.1%) responded to the questionnaire representing 14 European countries (Albania, Croatia, Denmark, Germany, Greece, France, Italy, Netherlands, Norway, Romania, Spain, Sweden, Switzerland, Turkey, and the United Kingdom), with 1–13 per country, and 3 non-European countries (New Zealand, Singapore, and Australia; Figure 1). Median age was 44 years (interquartile range = 38–51 years). The participants were working in an infectious diseases (67%, *n* = 43/64) or clinical microbiology (30%, *n* = 19/64) department. Sources of medical information for their practice were national guidelines alone for 8% (*n* = 5/64) of them, international guidelines only for 11% (*n* = 7/64), and an association of both for 64% (*n* = 41/64). TB specialist opinion was consulted, alone or together with other sources, by 50% (*n* = 32/64) of respondents. Among international recommendations, the main source was the WHO guidelines for 34% of respondents (*n* = 15/44).

### Microbiological TB Diagnosis

For the microbiological diagnosis of pulmonary TB, 97% (*n* = 62/64) of respondents used to collect at least one sputum in the early morning on an empty stomach, with 19% (*n* = 12/64) and 81% collecting two or three samples, respectively. The two remaining responders collected induced or spontaneous sputum at admission or randomly timed sputum sample. Multiple samples are usually taken during 3 consecutive days (58%, *n* = 37/64), or 2 (33%, *n* = 21/64) or within the same day (17%, *n* = 11/64). If the patient could not produce sputum, the alternatives were induced sputum (66%, *n* = 42/64), bronchoscopy aspirate (59%, *n* = 38/64), and gastric aspiration (27%, *n* = 17/64).

A rapid molecular test was performed systematically by 41/64 (64%) respondents, on one (31%, *n* = 14/45) or multiple respiratory specimens (33%, *n* = 15/45). In case of strong suspicion of pulmonary TB with negative sputum smears, 16/64 (25%) respondents would start treatment, 11/64 (17%) would wait for the culture result before starting, and 44/64 (78.6%) would perform additional diagnostic tests, namely, rapid molecular tests (66%), or bronchoscopy aspirate testing (61.3%). In case of positive sputum smear, a rapid molecular test was performed to detect mutations conferring resistance to rifampicin by 84% (*n* = 52/64), to isoniazid by 29% (*n* = 18/62), and to other drugs by 7% (*n* = 4/62), whereas 10 (16%) did not perform any rapid molecular test. Regarding the type of mutation sought for isoniazid resistance, among the 26 microbiologists who gave an answer, 17 (65%) perform rapid molecular tests to detect mutations in both *katG* gene and the *inhA* promoter, and four only in the *katG* gene (15%). Eight respondents (31%) were looking for genotypic resistance to other drugs besides rifampicin and isoniazid, with five of them performing whole-genome sequencing.

In the case of positive culture, of 61 respondents, 38 (62%) were asking for molecular detection of rifampicin resistance mutation, 24 (39%) for an isoniazid resistance mutation, and 9 (15%) for resistance mutations to other drugs, whereas 23 (38%) were not looking for genotypic resistance.

For the diagnosis of extrapulmonary TB, nucleic acid amplification test (NAAT), and measurement of adenosine deaminase (ADA) were performed in 85% (*n* = 52/61 responses) and 40% (*n* = 25/62 responses) of cases, respectively. ADA was performed mainly for the diagnosis of pleural effusion (96%, *n* = 24/25), but also on the cerebrospinal fluid (CSF) (40%, *n* = 10/25) in case of suspicion of TB meningitis and on peritoneal fluid (24%, *n* = 6/25). Sputum smear was repeated after 2 and 6 months of treatment by 53/63 (83%) and 37/63 (58%) participants, respectively. Overall, interferon-γ release assays (IGRAs) and tuberculin skin testing (TST) were performed by 31% (*n* = 20/64) and 23% (*n* = 15/64) of respondents, respectively, even for the diagnosis of active TB.

### Isolation Practices

Overall, 23% (*n* = 15/63) of respondents used a conventional single room to isolate pulmonary TB patients, whereas 77% (*n* = 50/63) reported using a negative-pressure room whatever the drug susceptibility profile of the strain. Regarding the duration of isolation for pulmonary TB with smear-positive sputum, 23/64 (36%) respondents adopted a standardized duration of 2 to 3 weeks, whereas 23/64 (36%) reported waiting for the sputum smear-negative conversion. In case of patients hospitalized with a suspicion of pulmonary TB but negative sputum smears and pending culture results, 18/64 (28%) of the respondents did not isolate the patient. Patients with a positive sputum smear were allowed to leave the hospital by 75% (*n* = 48/64) of respondents, in the following cases: if they agreed to wear a mask (*n* = 31/48, 65%), if there were no other people at home (*n* = 39/48, 81%), and in the absence of children (*n* = 29/48, 60%) or of immunocompromised individuals (*n* = 36/48, 75%) at home.

### TB Treatment

A majority of respondents (81%, *n* = 52/64) used the standard first-line treatment with isoniazid, rifampicin, pyrazinamide, and ethambutol for 2 months, followed by 4 months of treatment with isoniazid and rifampicin; but 14% (*n* = 9/64) of respondents offered an initial treatment without ethambutol. Fluoroquinolones were never prescribed as part of first-line treatment by 29/61 (45%) of respondents; conversely, 5/61 (8%) and 26/61 (41%), respectively, used them under certain conditions such as bone TB or suspicion of resistance to isoniazid. Overall, 30% (*n* = 19/63) of respondents reported to discontinue ethambutol treatment in the absence of mutations conferring resistance to isoniazid. Among respondents, 89% (*n* = 57/63) systematically gave B_6_ vitamin therapy. With regard to treatment monitoring, 50% (*n* = 32/62) of respondents performed an ophthalmology assessment during the first 2 months of ethambutol treatment. Overall, 50% (*n* = 32/64) of respondents never performed therapeutic drug monitoring for rifampicin and isoniazid, 17% (*n* = 11/64) routinely performed it, and 23% (*n* = 15/64) performed it only in specific cases (i.e., HIV infection, overweight, renal insufficiency). For neuromeningeal TB cases, 80% (43/54) of respondents added corticosteroids to the TB treatment for a variable duration, from 2 (10%, *n* = 4/40 respondents) to 12 weeks (5%, *n* = 2/40). The measurement of CSF pressure and repeated lumbar punctures were performed by 51% and 46% of the 59 respondents, respectively. In case of HIV coinfection, the most frequently used antiretroviral drugs were dolutegravir 50 mg twice a day (BID) (60%, *n* = 29/48 respondents), efavirenz 600 mg daily (35%, *n* = 17/48), a protease inhibitor with rifabutin (17%, *n* = 8/48), raltegravir 800 mg BID (15%, *n* = 7/48), raltegravir 400 mg BID (13%, *n* = 6/48), and efavirenz 800 mg daily (6%, *n* = 3/48). All respondents offered preventive treatment to HIV-positive patients with latent TB infection, using the following: isoniazid for 6 months (84%, *n* = 43/51 respondents), isoniazid and rifampicin for 3 months (24%, *n* = 12/51), rifampicin for 3–4 months (24%, *n* = 12/51), or isoniazid plus rifapentine for 3 months (4%, *n* = 2/51). To optimize adherence and treatment support, clinicians used an interpreter (56%, *n* = 36/64), a nurse specialized in therapeutic education (48%, *n* = 31/64), systematic Directly Observed Therapy (DOT) (47%, *n* = 30/64), or hospitalization for the full duration of treatment (28%, *n* = 18/64). As an alternative to hospitalization, 39/64 (61%) offered a DOT at home with a nurse.

## Discussion

TB continues to be a priority public health challenge in high-income countries. While EU/EEA countries adopted the key principles of TB control and elimination through the Europe-specific consensus-based documents born within the Wolfheze initiative and subsequent documents, a uniform set of guidelines summarizing essential standards to guide European clinicians and health care workers was developed only in 2012 (7, 8). Both International Standards for Tuberculosis Control (ISTC) and ESTC prescribe a widely accepted level of TB care, to guide all health care providers and clinicians, both public and private, in achieving optimal standards in managing individuals who have active TB, latent TB infection, or signs and symptoms compatible with the disease. The standards are designed to complement existing national or international guidelines and are consistent with the WHO definitions and recommendations (15, 17).

Our survey of TB management shows that practical attitudes currently can differ in some respects.

Regarding the diagnosis of TB, the practices are relatively consensual with the continued use of sputum examination, although the modalities may differ in number or timeframe (19). It should be noted, however, that 13.8% of participants performed only one sputum examination, whereas the guidelines always recommend performing at least two sputum smears (Table 1). Alternative examinations when patients were unable to provide a sputum sample vary according to the centers with equally heterogeneous guidelines: IDSA/ATS/CDC recommendations favor sputum induction compared to bronchoscopy. NICE guidelines are the only ones to propose gastric lavage, whereas 25.9% of respondents use it in clinical practice (Table 1) (20, 21). We observed that almost all centers use NAAT, although these tests are not yet carried out systematically for the diagnosis of TB (only 62.1% of respondents) as recommended by the guidelines (19, 22, 23). Despite recommendations to look for mutations conferring resistance to rifampicin and isoniazid, 16% of participants did not perform rapid molecular detection testing for rifampicin resistance in case of smear-positive patients (24, 25). For extrapulmonary TB, a majority of participants (86.3%) in the survey performed several rapid molecular tests as recommended by international guidelines. In contrast, the measurement of ADA was not universally used (40%), and it was mainly confined to pleural fluid. This recommendation is not consistent across all guidelines, because the ADA is used only for the ATS/ IDSA/CDC and NICE guidelines for pleural fluid, peritoneal fluid, and CSF. The monitoring of sputum smear microscopy and sputum culture is only recommended in the ERS/ECDC and WHO guidelines at least at the end of completion of intensive phase and actually performed after 2 months of treatment by most participants (82.5%). Finally, it should be noted that almost a third and a quarter of the responders, respectively, used IGRA and TST tests for diagnosis, whereas this is not recommended in most guidelines. These tests cannot effectively differentiate active and latent TB, even though a phlyctenular TST could predict rather an active TB (26).

Concerning the prevention of the risk of transmission of TB, the ERS/ECDC and NICE guidelines clearly specify the need to isolate the patient in a negative-pressure chamber, whereas 22% of the participants did not (14, 18). The isolation duration of contagious patients remains controversial, with 36% of respondents having adopted standardized isolation duration of 2–3 weeks, like the guidelines of NICE and the French Hospital Hygiene Society (14, 16); 43% prefer to wait for sputum smear conversion, as recommended by WHO and ATS/IDSA/CDC guidelines (13, 15). It should be noted that the conditions for patients discharged from health care facilities remains poorly studied, and this lack of evidence is reflected in the international recommendations from academic societies.

Regarding the treatment of TB, the initial treatment with four drugs is highly consensual between the respondents and the different recommendations; however, 14% still prescribed an initial treatment with only three drugs (respondents from different countries). The role of rapid molecular tests for resistance to isoniazid in guiding the withdrawal of ethambutol from the intensive phase treatment is yet unclear. The role of fluoroquinolones in first-line treatment, pyridoxine prophylaxis, CSF pressure measurement, and therapeutic drug monitoring should be better defined with additional studies. Similarly, monthly ophthalmologic monitoring of ethambutol patients is recommended only by the North American ATS/IDSA/CDC guidelines (13). On the other hand, the use of corticosteroids in the treatment of central nervous system TB is well established in the literature and among the various guidelines, whereas 20% of the participants in our survey did not routinely prescribe corticosteroids. Among HIV-positive patients, it is well established that the duration of TB treatment should be identical to that of HIV-negative patients. The importance of managing drug–drug interactions is also well emphasized in the different guidelines, although there is no clear recommendation on which antiretrovirals should be used in combination with rifampicin. Counseling is mostly developed in WHO guidelines, which target low-resource countries. The collaboration of the interpreter and the nurse in therapeutic education is still infrequent for the stakeholders of our study: 57 and 50%, respectively, whereas most European countries report increasing TB rates among newly arrived migrants (27, 28).

### Analysis of Differences in Practice

It is difficult to analyze the variability of practices observed between the different respondents. There may be variability between countries due to different local recommendations but also a disparity of human or economic resources. The availability of medications, funding of TB programs, laboratory practice, staff availability, and TB epidemiology can differ between countries, but this phenomenon is probably lower in high-resource European countries than on other continents. Furthermore, in our survey, 7% of respondents used only local recommendations for TB care management, whereas 78% also relied on international recommendations, which should also limit the variability between countries. It must also be emphasized that interindividual variability within the same country is possible. Although the respondents are among the TB experts for each country, their management practice cannot be fully representative of their respective countries. For instance, the practice survey conducted in France in 2013 with specialists in infectious diseases, pneumology, and internal medicine showed that different doctors often had very different habits concerning the treatment of TB or the isolation of active TB (29). Finally, the heterogeneity of practices can also be partially explained by the heterogeneity between the different national or international recommendations, as we have seen above. This lack of consistency across the different guidelines, as it is the case, for example, with regard to isolation practices, can be confusing for the clinician.

The ISTC defined the essential level of care for managing patients who have or are presumed to have TB or are at increased risk of developing the disease. In a high-resource setting, such as the EU/EEA, higher standards of care can be attained with regard to TB diagnosis, prevention, and treatment. On this basis, the ESTC was published in 2012 as standards specifically tailored to the European setting. Since the publication of the ESTC, new scientific evidence has become available, and therefore, the standards were reviewed and updated in 2017. Despite these new standards tailored to the EU setting, the harmonization of practices needs to be further improved. This is conditioned by a better standardization of national and international recommendations, a wider communication with people in charge of TB, and complementary studies to increase the level of evidence on controversial aspects of TB care. If we take the example of the joint ERS and ESCMID guidelines on the management of infections due to nontuberculous mycobacteria, which are being updated, collaboration between the various European academic societies would probably be beneficial.

Our study has several limitations. First, our sample is too small to be representative. Second, the responders were selected within the ESGMYC group, with mainly microbiologists and infectious disease specialists. Therefore, this sample does not include a lot of pulmonary physicians, although they are also very involved in TB care. Third, as mentioned above, the management practice of the responders could not be fully representative of their respective countries. Nevertheless, we think that this survey gives an interesting overview of different practices in Europe and highlights a heterogeneity in the management of TB that should be confirmed by a study with larger sample. In conclusion, WHO's international recommendations for TB management are focused on countries with high incidence. Recommendations that are more adapted to the European socioeconomic and epidemiological context have also been developed under the auspices of the ERS and the ECDC. This study shows that the management of TB is probably still heterogeneous within high-income countries, in particular for the prevention of transmission and the treatment. Concerning microbiological diagnosis, the practices seem to be better standardized, as rapid molecular testing takes an important role across most European centers. With the main objective of control and eventual elimination of TB, it seems necessary to better harmonize and disseminate the recommendations for the management of TB in Europe. European scholarly societies, and in particular ESCMID, should play a prominent role.

## Data Availability Statement

The datasets analyzed in this article are not publicly available. Requests to access the datasets should be directed to frederic.mechai@aphp.fr.

## Author Contributions

All authors participated in the writing and editing of the article, the analysis of the data as well as the dissemination that the investigation at the origin of this work.

## Conflict of Interest

The authors declare that the research was conducted in the absence of any commercial or financial relationships that could be construed as a potential conflict of interest.
