# Supplementary material for: Management of Tuberculosis: Are the Practices Homogeneous in High-Income Countries?
Source: Front Public Health. 2020 Sep 4;8:443. doi: 10.3389/fpubh.2020.00443 (PMC7509453; doi:10.3389/fpubh.2020.00443)
Supplement: Supplementary file 1 [file Data_Sheet_1.PDF]

European survey on the diagnosis and management of tuberculosis

ESGMYC_Mechaï & Cambeau

1. **Adresse e-mail** *

____

The main objective of this survey is to have a working base and an idea about our respective practices.

Identity of the participant:

2. **Surname, First Name (facultative)**

____

3. **Country**

____

4. **Institution**

____

5. **Age (years)**

____

6. **What is your profession ?**
Une seule réponse possible.

- ◯ Internal Medicine
- ◯ Infectious Diseases
- ◯ Respiratory Medicine
- ◯ Clinical Microbiology
- ◯ Researcher
- ◯ Autre : ______________________________

7. **What information do you use to support your practice ?**
Plusieurs réponses possibles.

- [ ] National Guidelines
- [ ] International Guidelines
- [ ] TB specialist opinion
- [ ] Autre : ______________________

8. **If yes, name or web-link of the guidelines and/or TB specialist**

____

Tuberculosis Diagnosis

9. **For a suspicion of active pulmonary tuberculosis, which investigations would you usually request?**
Plusieurs réponses possibles.

- [ ] Early morning sputum sample
- [ ] Autre : ______________________

10. **If the patient can't produce sample**
Plusieurs réponses possibles.

- [ ] sputum induction
- [ ] gastric aspiration
- [ ] bronchoscopy with BAS/BAL
- [ ] Autre : ______________________

11. **How many times would you repeat your investigation(s) (sputum or gastric sampling…) ?**
Une seule réponse possible.

- ( ) Twice
- ( ) Three times

12. **When do you do your investigations ?**
Plusieurs réponses possibles.

- [ ] On the same day
- [ ] Over 2 days
- [ ] Over 3 days

13. **Do you always ask/perform nucleic acid amplification test (NAAT)* for M. tuberculosis complex on the sputum**

* NAAT such as Xpert MTB-Rif, GenoType MTBDR, BDX, Anyplex, etc.…
Une seule réponse possible.

- Yes
- No

14. **If yes:**

Plusieurs réponses possibles.

- [ ] On one sample
- [ ] On the first sample
- [ ] On several samples

15. **If several samples with NAAT, how many ?**

____

16. **In the case of a strong suspicion of pulmonary TB, with negative smears and no clinical emergency, do you ?**

Plusieurs réponses possibles.

- [ ] Start the treatment
- [ ] Wait for the culture results before starting the treatment
- [ ] Perform a bronchoscopy
- [ ] Ask/perform NAAT

17. **If NAAT answered for negative smears, when ?**

Plusieurs réponses possibles.

- [ ] - on morning sputum sample
- [ ] - on Bronchial aspirate or alveolar
- [ ] - on Post-bronchoscopy sputum sample

Positive SMEAR

18. **In your usual practice, for SMEAR-positive samples, do you SYSTEMATICALLY ask for ?**

Plusieurs réponses possibles.

- [ ] molecular detection of rifampicin resistance (rpoB mutation)
- [ ] molecular detection of INH resistance
- [ ] Other resistance genes
- [ ] I don't ask for resistance genes

19. **If you ask for RMP resistance, which technique specifically ?**
Une seule réponse possible.

- ( ) GenXpert
- ( ) Autre : ____

20. **If you ask for INH resistance, which mutation ?**
Plusieurs réponses possibles.

- [ ] katG
- [ ] promoter inhA
- [ ] Autre : ____

21. **If you ask for INH resistance, which technique specifically ?**

____

22. **If you ask SYSTEMATICALLY other resistance genes than RMP & INH, for which antibiotics ?**

____

23. **If you ask SYSTEMATICALLY other resistance genes than RMP & INH, which technique ?**
Plusieurs réponses possibles.

- [ ] amplicon Sanger PCR
- [ ] molecular detection of many resistance markers by NGS sequencing
- [ ] • amplicon next generation sequencing (NGS)
- [ ] • Whole genome sequencing (WGS)

24. **if WGS, could you please give the type of WGS sequencer (Miseq, Hiseq, Minion, PacBio, others..) :**

____

Positive CULTURE

25. **In your usual practice, for CULTURE-positive samples, do you systematically ask for:?**
Plusieurs réponses possibles.

- [ ] molecular detection of rifampicin resistance (rpoB mutation)
- [ ] molecular detection of INH resistance
- [ ] Other resistance genes
- [ ] I don't ask for resistance genes

26. **If you ask for RMP resistance, which technique specifically ?**
Une seule réponse possible.

- ( ) GenXpert
- ( ) Autre : ____________________

27. **If you ask for INH resistance, which mutation ?**
Plusieurs réponses possibles.

- [ ] katG
- [ ] promoter inhA
- [ ] Autre : ____________________

28. **If you ask for INH resistance, which technique specifically ?**

____

29. **If you ask SYSTEMATICALLY other resistance genes than RMP & INH, for which antibiotics ?**

____

30. **If you ask SYSTEMATICALLY other resistance genes than RMP & INH, which technique ?**
Plusieurs réponses possibles.

- [ ] amplicon Sanger PCR
- [ ] molecular detection of many resistance markers by NGS sequencing
- [ ] • amplicon next generation sequencing (NGS)
- [ ] • Whole genome sequencing (WGS)

31. **if WGS, could you please give the type of WGS sequencer (Miseq, Hiseq, Minion, PacBio, others..) :**

____

Microbiological monitoring

32. **Do you systematically check culture negative conversion for MTb in sputum samples after two months of treatment ?**

Une seule réponse possible.

- Yes
- No

33. **Do you systematically check culture negative conversion for MTb in sputum samples after 5 or 6 months of treatment ?**

Une seule réponse possible.

- Yes
- No

34. **Do you ask/perform NAAT for non-pulmonary samples/sites ?**

Une seule réponse possible.

- Yes
- No

35. **If yes, which technique for which site ?**

ADA

36. **Do you use ADA (adenosine desaminase) levels ?**

Une seule réponse possible.

- Yes
- No

37. **If yes for ADA use, when ?**
Plusieurs réponses possibles.

- [ ] for pleural effusions
- [ ] for CSF
- [ ] for peritoneal punction
- [ ] for nodes punction
- [ ] Autre : ____________________

TB Meningitis diagnosis

38. **For tuberculous meningitis, do you use?**
Plusieurs réponses possibles.

- [ ] Pressure of lumbar puncture
- [ ] Corticosteroids
- [ ] CSF follow up
- [ ] NAAT on CSF

39. **Which corticosteroid drug do you use and which dosage ?**

____

40. **How long do you use corticosteroids ?**
in weeks

____

41. **Which dosage of Rifampicin do you use for TB Meningitis ?**
in mg/Kg

____

IGRA & TST

42. **For active Tuberculosis diagnosis, do you use IGRAs Test ?**
Une seule réponse possible.

- ( ) Yes
- ( ) No

43. **If Yes, for which situation ?**

____

44. **For active Tuberculosis diagnosis, do you use TST (Mantoux) ?**
Une seule réponse possible.

- Yes
- No

45. **If Yes, which size would be of interest ?**
in mm

____

Tuberculosis Isolation

46. **In which kind of isolation room do you hospitalize the patients with active pulmonary confirmed tuberculosis ?**
Plusieurs réponses possibles.

- [ ] Classical single room
- [ ] Single room with negative pressure
- [ ] Autre : ______________________

47. **For patients with smear positive sputum samples, do you have a standardized duration of isolation ?**
Une seule réponse possible.

- Yes
- No

48. **If yes, what is the duration ?**
Number of days

____

49. **If you do not have a standardized duration of treatment, in what circumstances do you stop isolation ?**
Une seule réponse possible.

- Only when you know that sputum are smear negative
- Always at DAY 15
- After DAY15 regardless of sputum results if the patient is treatment compliant and there is favorable clinical evolution
- After resolution of cough
- If the number of bacilli at microscopy decreases
- Only if sputum culture is negative
- Autre : ______________________

50. **In case of smear negative patients with suspicion of pulmonary tuberculosis ?**
Plusieurs réponses possibles.

- [ ] Isolation only for patients hospitalized
- [ ] Isolation of every patient until negativization result of culture
- [ ] Isolation only if there are abnormalities on chest radiography
- [ ] No need for isolation

51. **Do you ever authorize patients with smear-positive sputum samples to return home ?**
Une seule réponse possible.

- ( ) Yes
- ( ) No

52. **If yes, under what conditions ?**
Plusieurs réponses possibles.

- [ ] With a mask
- [ ] Without a mask
- [ ] Despite other people present at home
- [ ] If no children at home
- [ ] If no immunosuppressed people at home

53. **Do you propose alternatives to hospitalization to treat some patients :**
Plusieurs réponses possibles.

- [ ] DOT with community nurses
- [ ] Follow up with other community organisations
- [ ] Treatment in a free outpatient clinic
- [ ] Autre : ____________________

Tuberculosis Treatment

54. **What is your standard treatment of tuberculosis ?**
Une seule réponse possible.

- ( ) 2HRZ/4HR tritherapy
- ( ) 2HRZE/4HR quadritherapy
- ( ) Autre : ____________________

55. **When do you prescribe fluoroquinolones as first line treatment ?**
Plusieurs réponses possibles.

- [ ] Never
- [ ] Suspicion of isoniazid resistance
- [ ] Bone tuberculosis
- [ ] Autre : ____________________

56. **Concerning the treatment : Do you stop ethambutol if no isoniazid resistance mutation is detected before phenotypic susceptibility testing**
Une seule réponse possible.

- ( ) Yes
- ( ) No

57. **Do you usually give B6 vitamin with isoniazid treatment:**
Une seule réponse possible.

- ( ) Yes
- ( ) No
- ( ) Under conditions

58. **Which conditions**

____

59. **Do you request an ophthalmology exam for patient taking ethambutol treatment during the first 2 months of treatment ?**
Une seule réponse possible.

- ( ) Yes
- ( ) No

60. **Do you check drug blood levels following treatment with rifampicin and isoniazid ?**
Plusieurs réponses possibles.

- [ ] Never
- [ ] Always
- [ ] If HIV co infection
- [ ] If renal failure
- [ ] If excess weight
- [ ] Autre : ____________________

61. **In case of HIV-TB coinfection, what would you recommend in your country for HIV treatment ?**
Plusieurs réponses possibles.

- [ ] efavirenz 600mg
- [ ] efavirenz 800 mg
- [ ] raltegravir 400 mg bid
- [ ] raltegravir 800 mg bid
- [ ] dolutegravir 50 mg bid
- [ ] protease inhibitor with rifabutin
- [ ] Autre : ____________

62. **For HIV co-infection patients with positive test (TST and/or IGRAs) for presumed latent infection with M.tuberculosis but do not have active tuberculosis, which is the regimen used**
Plusieurs réponses possibles.

- [ ] INH for 6 months
- [ ] RFP for 3-4 months
- [ ] INH+RFP for 3 months
- [ ] INH+Rifapentine for 3 months
- [ ] No preventive treatment

63. **How do you manage treatment compliance of active tuberculosis (multiple responses are possible)**
Plusieurs réponses possibles.

- [ ] Use of interpreter
- [ ] Use of therapeutic education nurse
- [ ] DOT systematically
- [ ] DOT if non adherence
- [ ] Hospitalization during all the treatment if non adherence

Fourni par
Google Forms
